# Serological and Molecular Studies of a Novel Virus Isolate Causing Yellow Mosaic of Patchouli [*Pogostemon cablin* (Blanco) Benth]

**DOI:** 10.1371/journal.pone.0083790

**Published:** 2013-12-30

**Authors:** Mohammad Zaim, Ashif Ali, Jomon Joseph, Feroz Khan

**Affiliations:** 1 Crop Protection Division, CSIR-Central Institute of Medicinal and Aromatic Plants, Lucknow, India; 2 National Centre for Cell Science, Ganeshkhind, Pune, India; 3 Plant Biology Division, CSIR-Central Institute of Medicinal and Aromatic Plants, Lucknow, India; University of Texas Medical Branch, United States of America

## Abstract

Here we have identified and characterized a devastating virus capable of inducing yellow mosaic on the leaves of Patchouli [*Pogostemon cablin* (Blanco) Benth]. The diagnostic tools used were host range, transmission studies, cytopathology, electron microscopy, serology and partial coat protein (CP) gene sequencing. Evidence from biological, serological and sequence data suggested that the causal virus belonged to genus *Potyvirus*, family *Potyviridae*. The isolate, designated as *Patchouli Yellow Mosaic Virus (PaYMV*), was transmitted through grafting, sap and the insect *Myzus persicae* (Sulz.). Flexuous rod shaped particles with a mean length of 800 nm were consistently observed in leaf-dip preparations from natural as well as alternate hosts, and in purified preparation. Cytoplasmic cylindrical inclusions, pinwheels and laminar aggregates were observed in ultra-thin sections of infected patchouli leaves. The purified capsid protein has a relative mass of 43 kDa. Polyclonal antibodies were raised in rabbits against the coat protein separated on SDS – PAGE; which were used in ELISA and western blotting. Using specific antibodies in ELISA, *PaYMV* was frequently detected at patchouli plantations at Lucknow and Bengaluru. Potyvirus-specific degenerate primer pair (U335 and D335) had consistently amplified partial CP gene from crude preparations of infected tissues by reverse transcription polymerase chain reaction (RT-PCR). Comparison of the PCR product sequence (290 bp) with the corresponding regions of established potyviruses showed 78–82% and 91–95% sequence similarity at the nucleotide and amino acid levels, respectively. The results clearly established that the virus under study has close homology with *watermelon mosaic virus (WMV)* in the coat protein region and therefore could share a common ancestor family. Further studies are required to authenticate the identity of *PaYMV* as a distinct virus or as an isolate of *WMV*.

## Introduction

Patchouli [*Pogostemon cablin* (Blanco) Benth]; (Synonym *Pogostemon patchouli* Pellet.Vat. suavis Hook)], family Labiatae from tropical Asia is cultivated in Malaysia and Indonesia. It is now extensively cultivated in China, Indonesia, India, Malaysia, Mauritius, Taiwan, the Philippines, Thailand, and Vietnam. In India, about 30 species of patchouli have been reported. Following species of patchouli; *Pogostemon cablin*, P. commosum, P. hortensis, P. heyneasus and P. plectranthoides are cultivated for patchouli oil. It is an herbaceous perennial plant and the oil glands are present on its leaves. Patchouli oil is a viscous liquid, yellowish green to brown in colour and has powerful and persistent fragrance. Patchouli resinoid extracted from the leaves is used in soaps and perfumes. Patchouli oil is also used in cosmetics, tobacco and incense products. The therapeutic properties of patchouli include: anti-depressant, anti-inflammatory, antiseptic, aphrodisiac, astringent, carminative, diuretic, febrifuge, fungicide, insecticide, sedative and tonic. It also possesses many antifungal and bacteriostatic properties [1], [2]. Indonesia accounts for more than 80% of estimated annual world production and till 1996 it was the major producer and exporter of patchouli oil.

Some viruses naturally infecting patchouli have already been completely or partially characterized (Table 1). *Patchouli virus X* (*PatVX*) [3], *Patchouli mild mosaic virus* (*PaMMV*) [4] and *Peanut stripe virus* (*PStV)* [5] have been characterized and sequenced. There are other reports in which viruses either have been characterized at the preliminary level or published as disease reports; e.g., *potexvirus* [6], [7], *patchouli mottle virus* (*PaMoV*) [4], *Pepper ring spot virus* (*PRV*) [8], *Patchouli mosaic virus* (*PaMV*) [9], *Tobacco necrosis virus* (*TNV*) - a presumed *Rhabdovirus* [10] and yellow mosaic of Patchouli [11], [12], [13].

**Table 1:** Viruses characterized from patchouli plant.

Name of virus	Geographical location	Taxonomic position	GenBank Accession Number (NCBI, USA)
*Patchouli virus X (PatVX)*	Brazil	Alphaflexiviridae, *potexvirus*	NA
*Patchouli mild mosaic virus (PaMMV)*	Japan	Secoviridae, *Fabavirus*	NC_003975(RNA 1), AB011007 (RNA 2)
*Patchouli mottle virus(PaMoV)*	Japan	Potyviridae, *Potyvirus*	NA
*Pepper ring spot virus (PRV)*	Brazil	Virgaviridae, *Tobravirus*	NA
*Peanut stripe virus (PStV)*	India	Potyviridae, *Potyvirus*	AJ851894.
*Patchouli mosaic virus (PaMV)*	Brazil		NA
*Tobacco necrosis virus (TNV)*	Brazil	Tombusviridae, *Necrovirus*	NA
*Patchouli yellow mosaic virus (PaYMV)*	India	Potyviridae, *Potyvirus*	JQ 723729 (current study)

NA refers sequences not available in GenBank nucleotide database (NCBI, USA).

According to the latest scheme of classification, the family *Potyviridae* consists of 6 genera based on their transmission; Fungi (*Bymovirus*), whiteflies (*Ipomovirus*), aphids (*Macluravirus, Potyvirus*) or mites (*Rymovirus, Tritimovirus*) [14]. *Potyvirus* is the largest among the six genera in the family *Potyviridae* [14], [15]. The number of viruses now recognized as potyviruses has expanded rapidly in the recent years to a current estimate of more than 175 definitive and possible members [16], [17]. These viruses are 720–850 nm in length and are transmitted by aphids. They can also be easily transmitted by mechanical means. The genome of the virus is linear, single-stranded positive sense monopartite or bipartite RNA of 8,500–12,000 nucleotides with a poly (A) tail at the 3′ end and probably a genome-linked protein (VPg) at its 5′ end. The genome or genome segments are translated into polyproteins, which are subsequently processed by virus encoded proteases into functional proteins.

**Figure 1:**
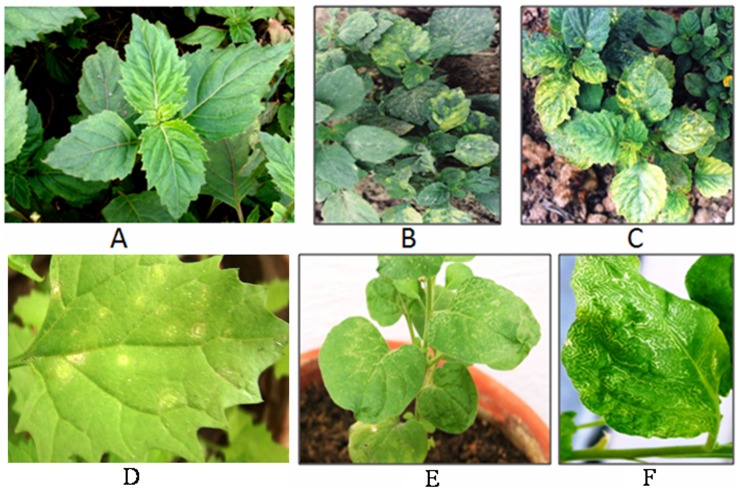
Symptomatology of *PaYMV*, healthy patchouli plant (A), initial stage of disease (B), mature stage of disease (C), chlorotic local lesions surrounded by ring on *C. quinoa* Wild. (D), ring spot & line pattern on *N. benthamiana*, initial stage (E) and mature stage of symptoms (F).

Patchouli (*Pogostemon cablin*) was consistently found to exhibit yellow mosaic symptoms every year at the experimental farm of CSIR (Council of Scientific & Industrial Research)-Central Institute of Medicinal and Aromatic Plants, Lucknow, Uttar Pradesh and at its Resource Centre, Bengaluru, India. Healthy plants showed dark green leaves (Fig. 1A) whereas symptoms of diseased plants included malformation of the leaves with yellow mosaic symptoms and overall retardation of plant growth (Fig. 1B & C). Almost all plants in the fields exhibited these disease symptoms. Considering the importance of the disease, studies were undertaken to identify the virus associated with the typical disease symptoms and characterize it at the serological as well as molecular levels. Our studies suggested that *Patchouli yellow mosaic virus* (*PaYMV*) was identified as the causative agent associated with the typical disease symptoms in patchouli and could be a new potyvirus or an isolate of *Watermelon mosaic virus (WMV)*.

## Results

### 2.1. Disease Symptoms

Leaves of infected plants of *P. cablin* from the field showed mottling followed by yellow spots and discolored patches of irregular shapes and sizes (Fig. 1B & C). The symptoms were relatively more pronounced with higher temperatures during the months of April to June, in North Indian conditions. The infected plants displayed highly stunted growth. In the case of severe infection, leaves exhibited intense yellow mosaic patches.

### 2.2. Host Range and Symptomatology

For host range trial, forty-two species from ten families were tested, Out of that very few were susceptible and showed systemic symptoms; some showed local lesions and a majority of them remained asymptomatic (Table 2), showing that *PaYMV* had restricted host range. Inconspicuous chlorotic lesions of 1–2 mm diameter appeared on *Chenopodium quinoa* Wild., 5–8 days post inoculation (dpi) (Fig. 1D). *C. amaranticolor* Cost & Ryne. produced necrotic local lesions (NLL) 5–8 dpi.

**Table 2:** Reaction of host range plants inoculated with *PaYMV*.

Virus reaction	Family	Scientific Name	External symptoms
Local symptoms	Amaranthaceae	*C.amaranticolor* cost & Ryne	NLL, CR
		*C. quinoa* wild	CLL
		*C. murale*	NLL
Systemic symptoms	Apocynaceae	*C.roseus*	Mt
	Asteraceae	*Z.elegans*	Ms
	Solanaceae	*N.benthmiana*	SM, LP, N
Symptomless	Amaranthaceae	*Beta vulgaris*	NS
	Brassicaceae	*Arabidopsis thaliana*(L.)Heynh	NS
	Cucurbitaceae	*Citrullus lanatus*	NS
		*Cucumis sativa L*	NS
		*C. melo*	NS
		*Cucurbita moschata*	NS
		*Lagenaria siceraria*	NS
		*Luffa cylindrical*	NS
	Solanaceae	*N.glutinosa*	NS
		*N.rustica*	NS
		*N.tobacum var.sansum*	NS
		*N.tobacum var white burley*	NS
		*N.tabacum CV CTRI special*	NS
		*N.tobacum var.xanthi*	NS
		*D.stramonium*	NS
		*D.inoxia*	NS
		*D.metel*	NS
		*P.minima*	NS
		*S.tuberosum*	NS
		*S.melongena*	NS
		*P.hybrida*	NS
	Malvaceae	*S.nigrum*	NS
		*Gossypium*	NS
	Cyperaceae	*H.esculentus*	NS
	Ranunculaceae	*C.esculentus*	NS
	Fabaceae	*H.niger*	NS
		*P.sativum*	NS
		*D.lablab*	NS
		*Psoralea corylifolia*	NS
	Asteraceae	*V.faba*	NS
		*Xanthium strumarium*	NS
		*Sonchus spc.*	NS
		*B.pilosa*	NS
	Apocynaceae	*H. annuus*	NS
		*C.gigantea*	NS
	Papaveraceae	*P.somniferum*	NS

NLL: Necrotic local lesion; CR: Chlorotic ring; CLL: Chlorotic local Lesion; SM: severe Mosaic; LP: Line Pattern; NS: No Symptoms; N: Necrosis: Ms: Mosaic Mt; Mottling.

*Nicotiana benthamiana* Domin, was found as diagnostic as well as good multiplication host, which produced initial mottling, mosaic symptoms on leaves with reduced lamina and downward curling of terminal leaves. The basal leaves produced typical ring spots between 7–9 days post inoculation (dpi), which converged into a line pattern (Fig. 1E, F) from 13–15 dpi under the temperature between 25–30°C. The infected plants showed at least two fold increase in shoot branching. In the case of severe infection, white patches appeared on the leaves, which had ring spots. The infected plants produced flower buds but failed to complete flowering.

### 2.3. Mode of Transmission and Virus Culture Maintenance

The virus was transmitted host to host by grafting and mechanically by grinding the infected young leaves in 0.1 M Phosphate buffer pH 7 containing 0.1% β-mercaptoethanol. Culture of the virus isolate was initiated by a single lesion from *Chenopodium quinoa* Wild. and maintained on *N. benthamiana* Domin. In insect transmission tests, RT-PCR of *M. persicae* Sulz. just after the probing onto *PaYMV* infected *N. benthamiana* Domin. using U335and D335 potyvirus-specific degenerate primers as described, gave amplification of about 300 bp. However no PCR amplification was seen in aphids which were collected just before the acquisition feeding (Data not shown). The aphid (*M. persicae* Sulz.) could transmit the virus into 3 healthy *N. benthamiana* plants out of 5 plants tested in non-persistent manner.

### 2.4. Virus Characterization

**Figure 2:**
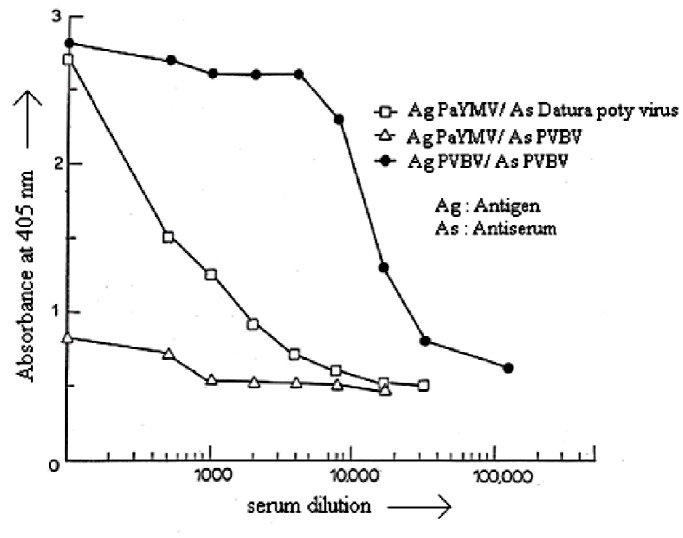
DAC-ELISA between *PaYMV* and heterologous antisera (as indicated).

**Figure 3:**
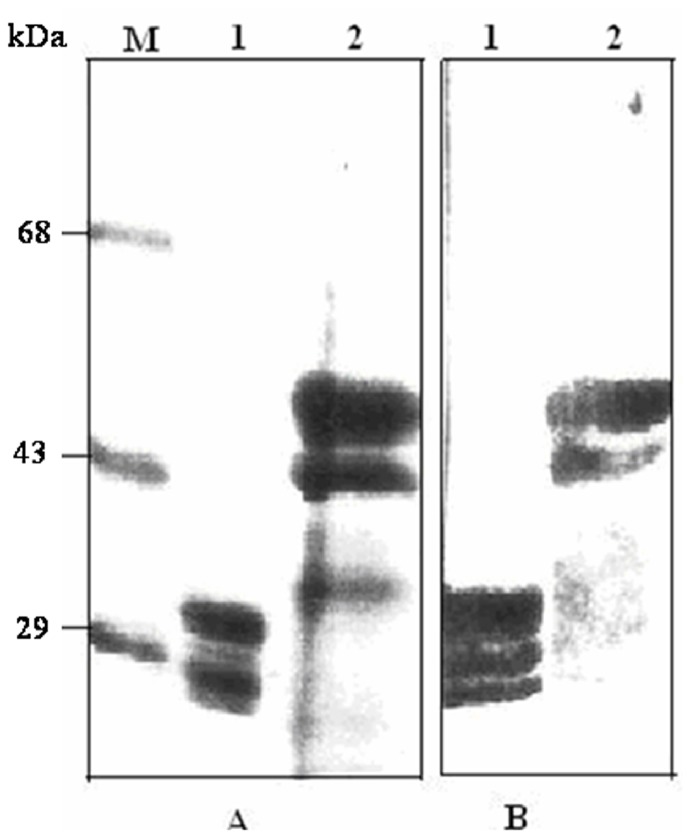
Sodium Dodecyl Sulphate – Polyacrylamide Gel Electrophoresis (A) and Western Blot (B) analyses using PVBV antiserum. Lane M = protein molecular weight markers, Lane 1 = purified *PMV-P* as positive control, Lane 2 = partially purified *PaYMV*.

*Datura potyvirus* and *pepper vein banding virus* (*PVBV*) antibodies were used for the cross reactivity test which showed the cross reaction of *PaYMV* with that of *PVBV* and *Datura potyvirus* (Fig. 2). The results of ELISA confirmed the presence of a possible potyvirus infecting *P. cablin*, as the antisera were group specific. The size of viral coat protein was determined to be about 43 kDa for *PaYMV*, whereas it was 34 kDa for *PMV-P* (Fig. 3A, lane 2), which is another *potyvirus* infecting the opium poppy crop available in the same field and has served as positive control. Western blot analysis (Fig. 3B) using the *PVBV* antiserum showed cross reaction of the antibodies with *PaYMV* and *PMV-P* (positive control). The lower molecular weight bands could be due to the degradation products of coat protein, as coat proteins of *potyviruses* have been shown to be highly susceptible to proteolytic degradation [18]. Interestingly, the CP of *PaYMV* showed a higher molecular weight compared to that of the other *potyviruses* tested. Complete sequencing of the CP gene is required to authenticate the exact molecular weight of the coat protein encoded by *PaYMV*.

**Figure 4:**
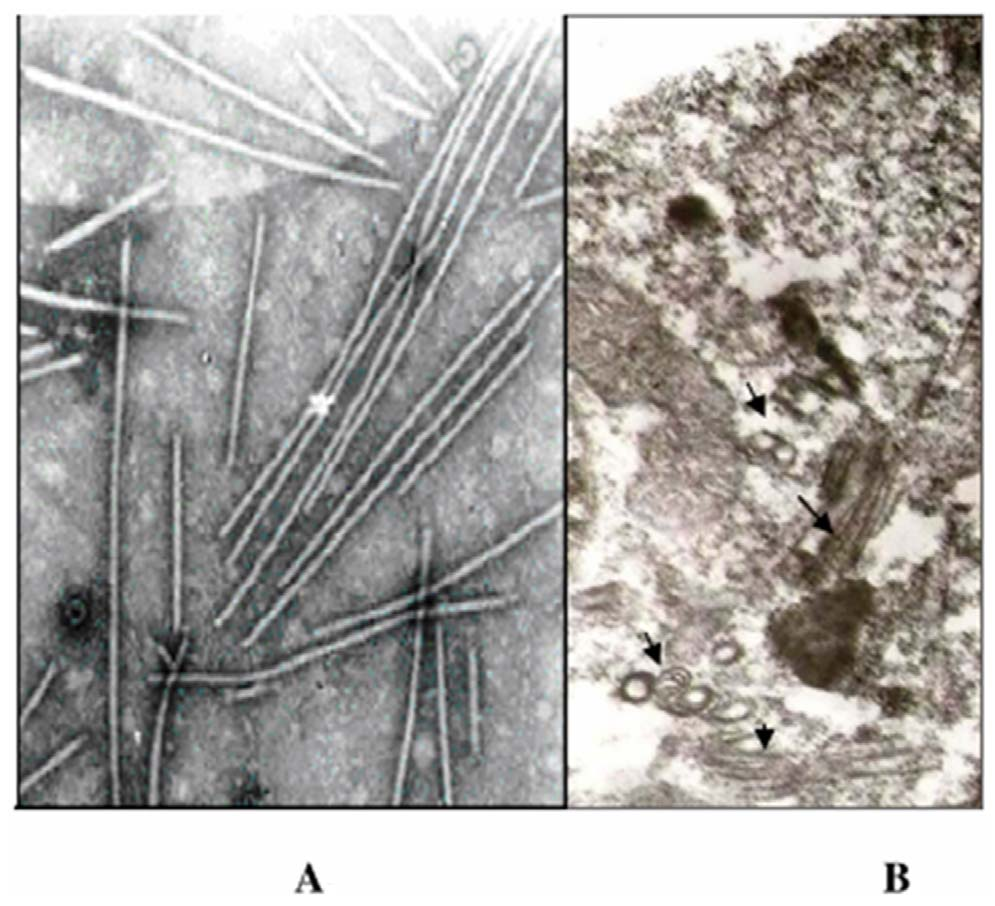
Electron micrographs showing flexuous rod particles in purified virus preparation (A) and Ultrathin sections showing Scrolls, cylindrical inclusions (B).

Purified virus preparation gave a yield of approximately 1.3 mg per 100 g of leaves. Interestingly, in purified virus preparations also flexuous rod shaped particles were observed as in leaf-dip preparations. Nodal length of *PaYMV* virus particles was calculated by measuring 100 virions, and was found to be around 800 nm in length and 11 nm in width (Fig. 4A). The main problem encountered in the isolation and purification of PaYMV was aggregation of virus particles which was reduced by the use of 1 M Urea, 1% Triton X-100, and 0.1% β-mercaptoethanol in the exraction and resuspension buffer. Negatively stained by 2% uranyle acetate Lleaf dip preparations of *N. benthamiana/P. cablin* (patchouli) infected with the *PaYMV* Isolates revealed flexuous rod shaped particles resembling those of potyviruses. In ultrathin-sections of infected cells, pinwheel, scroll like and laminated inclusions were detected in the cytoplasm (Fig. 4B). The pinwheel inclusions were also seemed to possess spiral arms.

### 2.5. Nucleotide Sequences Analysis and Molecular Characterization

**Figure 5:**
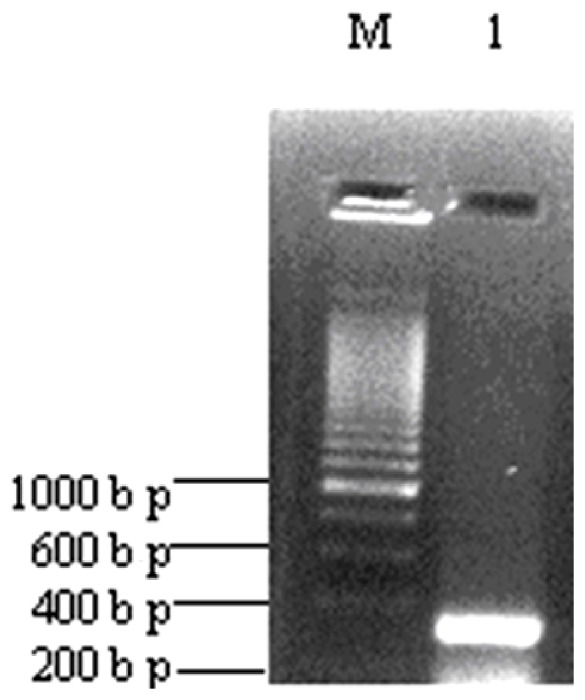
Amplification by RT-PCR of infected patchouli leaves. Lane M - 200 bp marker, Lane 1- amplified product.

A 290 bp DNA product was amplified using the total RNA prepared from *PaYMV* infected tissue by RT-PCR (Fig. 5) with the potyvirus-specific degenerate primer set as described. The amplicon was cloned, sequenced, and submitted to the GenBank database (NCBI, USA), with the accession number JQ723729. The deduced amino acid sequence contains 96 residues encoding part of the CP gene. Out of 96 residues, 88 were identical to that of *WMV* coat protein (EMB: CAD23063). When the gene sequence was compared with other known viral nucleotide sequences available in GenBank database (NCBI, USA), we found that *PaYMV* (GB: JQ723729) belongs to the family *Potyviridae*, and showed 81% sequence similarity with *WMV* Ard. Me (GB: JN166705).

### 2.6. Sequence Alignment and Phylogenetic Studies

**Figure 6:**
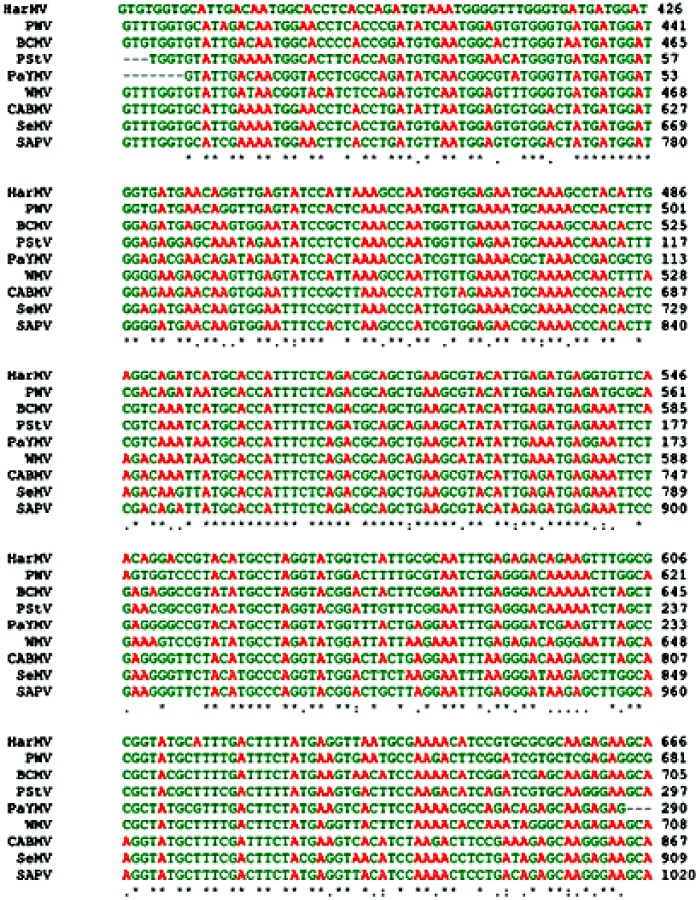
Multiple sequence alignment of coat protein (CP) gene of *PaYMV* (Acc. No.- JQ723729) and other potyviruses. Note: *WMV* (*Watermelon mosaic virus isolate* Ard. Me, Acc. No.- JN166705), *BCMV* (*Bean common mosaic virus* strain NL-4, Acc. No.- JN692258), *CABMV* (*Cowpea aphid-borne mosaic virus* isolate GSR1, Acc. No.- JF833417), *SeMV* (*Sesame mosaic potyvirus*, Acc. No- U90326), *SAPV* (*South African passiflora virus*, Acc. No.- S51666 ), *HarMV* (*Hardenbergia mosaic virus*, Acc. No- DQ898214), PWV (*Passion fruit woodiness virus*, Acc. No- JF427617), *PStV* (*Peanut stripe virus* Indian isolate, Acc. No.- AJ851894). Asterisks indicate identical nucleotides.

**Figure 7:**
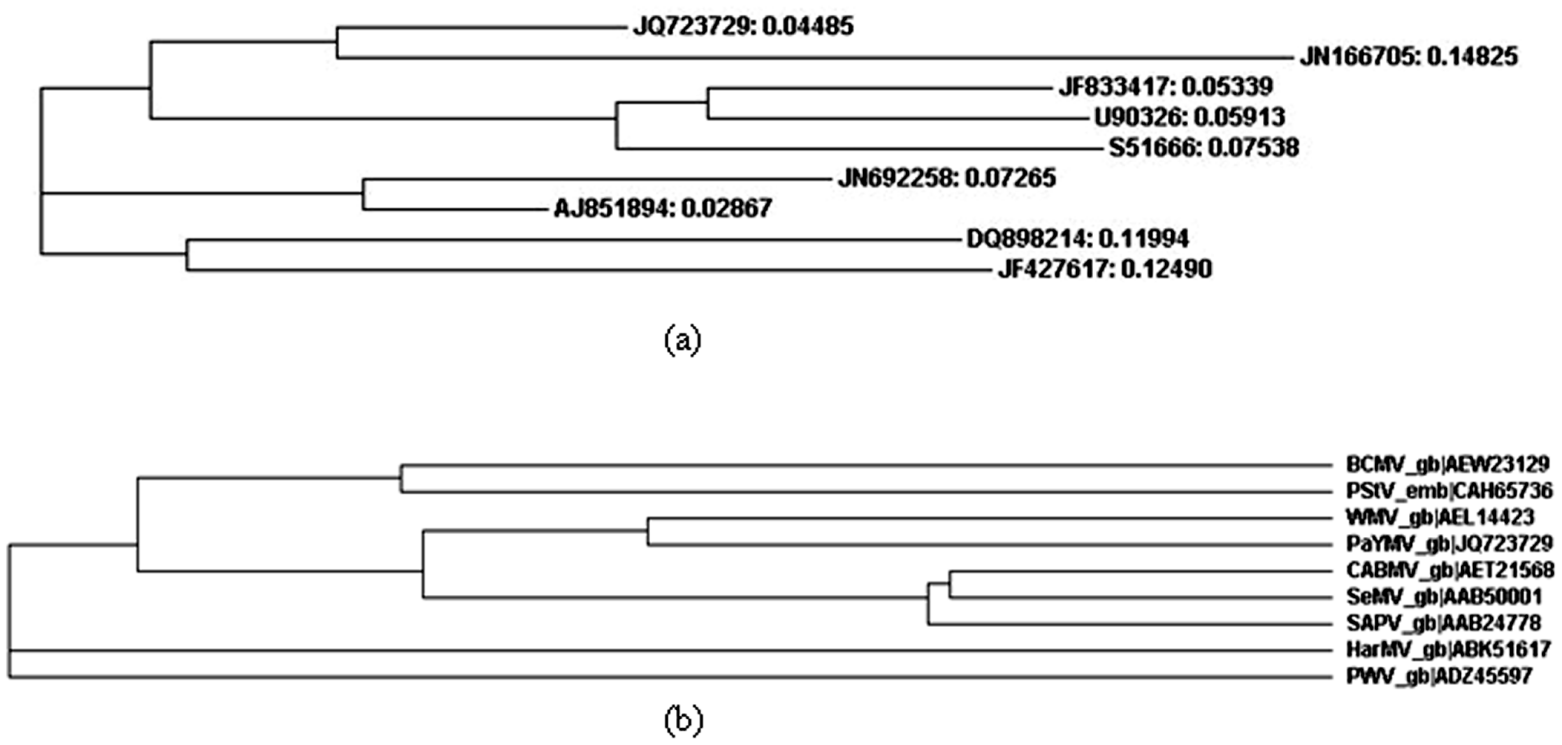
Phylogenetic tree constructed from the alignment of nucleotide and protein sequences of coat protein gene of *PaYMV*. (a) Phylogenetic tree based on nucleotide sequences of *PaYMV* (JQ723729; PaYMV_CP) and other potyviruses using neighbor-joining distance method. The numbers corresponding to gene accession indicate evolutionary distances. (b) Phylogenetic tree based on amino acid sequences of *PaYMV* (JQ723729; PaYMV_CP) and other potyviruses.

Results showed that the partial sequence of a PaYMV CP gene (PaYMV_CP) (GB: JQ723729) was similar to that of WMV Ard. Me (GB: JN166705) and therefore belonged to the same ancestral family clad of derived phylogenetic tree (Fig. 6 and 7A). Similarly in another clad, sequence of Cowpea aphid-borne mosaic virus isolate GSR1 polyprotein (GB: JF833417) showed higher sequence similarity with Sesame mosaic potyvirus polyprotein gene (GB: U90326), in comparison to South African passiflora virus SAPV polyprotein gene (GB: S51666). On the other hand, another clad of Bean common mosaic virus strain NL-4 coat protein gene sequence (GB: JN692258) showed higher nucleotide sequence similarity to a Peanut stripe virus partial coat protein gene (GB: AJ851894), therefore belongs to the same ancestral family, which is quite far from the PaYMV coat protein gene (GB: JQ723729). We found that more distantly located clad than PaYMV coat protein, was of the Hardenbergia mosaic virus isolate Sb-6 polyprotein gene (GB: DQ898214) and Passion fruit woodiness virus isolate CarW-3 coat protein gene (GB: JF427617), which showed higher sequence similarity with each other (Fig. 6 and Table 3). Similar evolutionary relationship pattern revealed in protein sequences based phylogenetic studies (Fig. 7B).

**Table 3:** Details of nucleotide and amino acid sequence similarity of *PaYMV* and other Potyviruses coat protein (CP) genes.

Sl. No.	Virus	Function	GenBank (NCBI) Accession No.	Gene length (nt.)	NCBI BLASTn statistics			GenBank (NCBI) Accession No.	Proteinlength (aa)	Identity	NCBI BLASTx statistics		
					Seq. Identity	E-value	Querycove-rage				Simil-arity	E-value	Query Cove-rage
1	BCMV	CP	gb JN692258	860	82%	1e-58	99%	emb CAD23061	109	91%	97%	3e-63	91%
2.	WMV	CP	gb JN166705	825	81%	6e-57	99%	emb CAD23063	109	92%	98%	1e-63	92%
3.	CAMV	PP (CP) gene	gb JF833417	1102	81%	2e-52	97%	gb AAP04516	284	92%	97%	2e-62	92%
4.	SeMV	PP (CP) gene	gb U90326	1296	79%	3e-44	98%	gb AAB50001	355	91%	97%	2e-63	99%
5.	SAPV	PP	gb S51666	1436	81%	4e-43	83%	gb ABQ95508	479	92%	97%	5e-62	99%
6.	HarMV	PP	gb DQ898214	825	80%	2e-42	87%	ref YP_004564598	273	91%	95%	2e-61	99%
7.	PWV	CP	gb JF427617	837	80%	7e-41	87%	gb ADZ45601	279	90%	96%	8e-62	99%

CP (Coat protein gene), PP (Poly protein gene), *BCMV* (*Bean common mosaic virus*), *WMV* (*Watermelon mosaic virus*), *CAMV* (*Cowpea aphid-borne mosaic virus*), *SeMV* (*Sesame mosaic potyvirus*), *SAPV* (*South African passiflora virus*), *HarMV* (*Hardenbergia mosaic virus*), *PWV* (*Passion fruit woodiness virus*).

## Discussion

Based on the size and morphology of virus particles, transmission by the insect *M. persicae*, serological reactivity to potyvirus to specific polyclonal antibodies in western blot analysis and ELISA, induction of pinwheel cylindrical inclusions, the *PaYMV* isolated from *P. cablin* was identified as a member of family *Potyviridae* and genus *Potyvirus*. The virus had narrow host range, which was restricted to families Apocynaceae, Asteraceae, Amaranthaceae. Susceptibility of *N. benthamiana* suggested that it might be *WMV-2* [19] as *WMV-1* is restricted to family Cucurbitacea only [20], [21] and *N. benthamiana* is diagnostically insusceptible host [20]. In addition to *WMV* Ard. Me, it also has shown significant homology with *Sesame mosaic potyvirus* (Fig. 6, Table 3) which is serologically related to *WMV-*2 and has *N. benthamiana* as the systemic host [21].

Identification of species was further confirmed by computational analysis. Partial gene sequence of PaYMV CP was compared with the nucleotide database of NCBI webserver using the BLAST tool. Results showed that query sequence was best pairwise aligned with gene sequence of *Bean common mosaic virus* NL-04, *Watermelon mosaic virus* Ard. Me and *Cowpea aphid-borne mosaic virus* isolate GSR1with similar sequence similarity and identity of 81–82%, but with variable BLAST score value i.e. 235 bits (127), 230 bits (124), and 215 bits (116), bits respectively. The BLASTn results indicated that the isolated sequence was corresponding to the sequence of potyvirus coat protein as expected.

For phylogenetic studies a separate data set of potyvirus coat protein gene sequences was prepared after removing the sequences of non-cultured and repeating strains from the BLAST result. The nucleotide level conservation was evaluated by comparing the gene sequences of *Bean common mosaic virus* NL-04, *Watermelon mosaic virus* Ard. Me, *Watermelon mosaic virus* isolate IR02-54, *Cowpea aphid-borne mosaic virus* isolate GSR1, *Sesame mosaic potyvirus* polyprotein (CP) gene, polyprotein [*South African passiflora virus*, Genomic RNA, 1436 nt], South African passiflora virus NIb and CP genes and Hardenbergia mosaic virus isolate Sb-6 polyprotein gene, Hardenbergia mosaic virus isolate Sb-15 polyprotein gene, *Peanut stripe virus* and *Patchouli yellow mosaic virus* cp gene (PaYMV_CP) through multiple sequence alignment analysis. Result of sequence alignment showed that conservation of nucleotides was much higher in *Bean common mosaic virus* strain NL-4 coat protein gene, and *Watermelon mosaic virus* isolate Ard. Me coat protein gene than *Cowpea aphid-borne mosaic virus* and *Passion fruit woodiness virus* isolate SP-1 coat protein gene (Fig. 6).

The PCR product sequence that represents a fragment of the coat protein gene of *PaYMV* was found to be similar to that of *Watermelon mosaic virus* from the nucleotide position 413 to 701, showing 81% sequence similarity (Fig. 6). Whereas results of BLASTx showed overall 98% similarity and 92% identity of *PaYMV* translated protein sequence from position 3 to 290 with *BCMV* protein sequence from position 5 to 100. Similarly, a fragment of the coat protein gene sequence of Patchouli *potyvirus* from nucleotide position 1 to 289 was found similar to that of *Bean common mosaic virus* coat protein gene sequence from position 413 to 701 and showing overall 82% sequence similarity, but due to the results of BLASTx which showed overall 97% similarity and 91% identity of *PaYMV* translated protein sequence from position 3 to 290 with *BCMV* protein sequence from position 5 to 100, it was considered less conserved than *Watermelon mosaic virus* coat protein gene (Fig. 7).

Earlier preliminary work has been done on viral diseases of patchouli except in three reports; *Patchouli Virus X* [3], *Patchouli mild mosaic virus* (*PaMMV*) [4] and *PStV* [5] where the virus was identified at the species level and partially or fully sequenced. The remaining studies were disease reports, three from India [11], [12], [13]. In this work, we developed antiserum against CP for the detection of *PaYMV,* which could be useful in ELISA, Ouchterlony double diffusion and western-blot tests. The study reported here suggests that *PaYMV* could be a distinct potyvirus or an isolate of WMV infecting Patchouli. Complete sequencing of the CP gene would be important to define the distinctness of the virus. Using this disease indexing tools, *PaYMV* can frequently be detected in patchouli **(** *Pogestemon cablin)* plants collected from different parts of India or other part of the world.

## Materials and Methods

### 4.1. Plant Materials

Test plants: Seedlings of test plants used in host range studies were raised in shallow pots of 50 cm and 30 cm in size and transplanted into earthen pots of 30 cm and 20 cm depending upon the growth rate of the test plants. Healthy seedlings were maintained in an insect proof glass house at the temperature ranging from 25°C to 35°C.

### 4.2. Mode of Transmission and Maintenance of Virus Culture

Culture of the virus isolate was initiated by a single lesion from *Chenopodium quinoa* Wild. and maintained on *Nicotiana benthamiana* Domin. Inoculum was prepared by grinding the infected young leaves in 0.1 M Phosphate buffer pH 7 with 0.1% β-mercaptoethanol. Periodic checks were made on diagnostic host *C. quinoa* Wild. to ensure the biological purity of the virus. The infected five patchouli stocks were grafted to the healthy scions to check the transmission of the causal agent by grafting. In another experiment, Aphids (*Myzus persicae* Sulz.) were used for the virus transmission. Virus free aphids were fed on *PaYMV* infected *N. benthamiana* Domin. plants for 24 hours. Reverse transcription-polymerase chain reaction (RT-PCR) has been performed using potyvirus specific degenerate primers as has been described to detect *PaYMV* in aphids before the probing and right after the probing according to the method used earlier [22]. After fasting for 4 hours aphids were transferred to 6 leaf stage *N. benthamiana.* Twenty-four hours later, aphids were killed by spraying 0.1% Demecron in one set. In another set of the plants, aphids were transferred to another set of healthy plants. This process was repeated for 10 days to test for non-persistent or persistent mode of virus transmission.

### 4.3. Host Range Studies

For host range studies many plants from diverse families were inoculated with the virus isolate. Sap prepared from leaves in 0.01 M Sodium phosphate buffer, pH7, was rubbed onto leaves pre-dusted with carborundum powder (600 mesh). The Leaves were then rinsed with distilled water, and plants were maintained in an insect proof glass- house for observations. Symptoms on both inoculated and upper, uninoculated leaves were recorded. Tests for latent infection were conducted by back–inoculation to either *C. amaranticolor Cost & Reyn* or *C. quinoa* Wild.

### 4.4. Electron Microscopy

Leaf- dip preparation was made by crushing the infected leaves patchouli and *N. benthamiana* and adsorbed on 400 mesh copper grid of pre-coated with carbon, which were negatively stained by 2% Uranyle Acetate. Similarly purified samples were also processed and examined under transmission electron microscope (JEOL 100 s).

### 4.5. Cytopathology

Young and vigorously growing leaves of patchouli plants with typical yellow mosaic symptoms were harvested when highly infectious and comparable leaves from healthy plant were also sampled. Pieces of leaf 1 cm^2^ were vacuum infiltrated with chilled 2.5% gluteraldehyde in 0.01 M Cacodylate buffer, pH 7.2 (v/v). After fixation in cold (about 2°C) for 1 h; 1 mm strips were cut avoiding tissue bordering on previously cut edges. The strips were blotted and washed in 0.01 M cacodylate buffer (pH 7.2). After blotting these pieces were transferred to cold 1% osmium tetraoxide (prepared in cacodylate buffer) till they become a Z-black. Thereafter leaf strips were processed for dehydration after thorough washing in distilled water in 30, 50, and 70% alcohol series for 30 minutes each at room temp. Dehydration was carried out for 15 min. at 90% alcohol and for 2×15 minute in 100% alcohol. A treatment of propylene oxide was given for 15 minutes to the dehydrated leaf strips. Infiltration was started taking a mixture of araldite and propylene oxide in 1∶1 ratio and thereafter into pure araldite. Araldite mixture was used as; Araldite-6005-5 g, DDSA –4.35 g.

### 4.6. Embedding: Araldite

The resin was prepared as reported [23].The ratio adjusted for the mixture: Araldite-6005-5 gm., DDSA −4.35 g, BDMA −0.11 ml. Moulds were kept for 60°C for 20 h.

### 4.7. Sectioning

Sections were cut with LKB ultra-tome Nova using glass knives. Only silvery sections were collected on 400 mesh grids.

### 4.8. Staining

Sections were stained with saturated aqueous uranyl acetate at room temperature for 6 hours and then rinsed in triple distilled water. All the sections further stained by 2% lead citrate [24] for 5 minutes at room temperature.

### 4.9. Purification of the Virus and Antisera Production

Young leaves with typical symptoms were harvested from the infected *N. benthamiana* at about 15–20 days after inoculation and grinded in 20 mM HEPES buffer (1∶3 wt/vol.), pH 7.2, containing 1 M Urea, 1% Triton X-100, 10 mM Di-ethyl Dithiocarbamate, and 0.1% β-mercaptoethanol. The extract was passed through cheesecloth and the filtrate was clarified with ice - cold 10% chloroform. The virus was precipitated by adding 6% polyethylene glycol 6000 and 0.2 M Nacl. The precipitate was suspended in resuspension buffer (0.02 M HEPES pH 7.2 containing 0.5 M urea and 0.02 M sodium sulfite), pelleted at 120,000 g for 3 h at 4°C in Sorvall ultracentrifuge Pro-80. The pellets were resuspended in resuspension buffer (20 mM HEPES pH 7.2 containing 0.5 M Urea and 0.02 M Sodium Sulfite) and loaded onto 10 to 40% preformed sucrose density gradient prepared in the same buffer and subjected to centrifugation at 92,500 g for 2.5 h in a SW28 rotor. A light scattering zone which appeared at about 1.4 cm from the bottom of the tube was collected and subjected to centrifugation at 120,000×g for 3 h. The final pellet was resuspended in a minimum volume of resuspension buffer and absorbance was taken in 260 and 280 nm. The purified virus preparation was observed under JEOL 100 s transmission electron microscope.

### 4.10. Production of Polyclonal Antiserum

Antiserum was produced in rabbits against the coat protein of PaYMV separated by SDS gel electrophoresis, by giving a series of intradermal injections. 500 µg of the virus was used in complete adjuvant for the first injection and 250 µg of the virus in incomplete adjuvant for three subsequent injections at 1 week intervals. The presence of PaYMV specific antibodies in the serum was tested by direct antigen coating – ELISA (DAC- ELISA) and western blotting, which confirmed the presence of respective antibodies in serum.

### 4.11. Virus Detection using an Enzyme Linked ImmunoSorbent Assay (ELISA)

The virus was initially identified serologically by direct antigen coating (DAC-ELISA as described [25]. Partially purified preparation of *PaYMV* was used as antigens which were pre-coated with100 ng/well in Polystyrene microtitre plates. *Pepper vein banding virus* (*PVBV*), which was a well characterized potyvirus specific polyclonal antibodies [26] were used to know the serological relationship. PVBV antigen in crude extract was used as positive control. Polyclonal antiserum against *PVBV* was used in serial double dilutions of 1∶100, 1∶1000, 1∶2000, and 1∶4000 up to 1∶164000. The binding of antibodies in the serum was assayed with goat anti-rabbit immunoglobulin (Ig) conjugated to alkaline phosphatase (sigma), the absorbance were read at 405 nm in a Bio- Tek auto reader EL311.

### 4.12. Coat Protein Analysis and Western Blotting

The molecular weight of viral coat protein was estimated by discontinuous SDS- PAGE (sodium dodecyl sulfate polyacrylamide gel electrophoresis) using a standard protocol [27]. The viral coat proteins from partially purified *PaYMV* were separated on 12% polyacrylamide gel and transferred onto nitrocellulose membrane. The blot was probed with polyclonal antiserum against *PVBV*.

### 4.13. Total RNA Extraction and RT – PCR

Total RNA was extracted from test plant *N. benthamiana* leaves sap inoculated with the virus and *M. persicae* Sulz. (just before the acquisition feeding and right after the acquisition feeding) using TRIzol® reagent (Invitrogen #15596-018 USA) according to the manufacturer’s instructions. After precipitating with ethanol total RNA was resolubilized in RNase free water. Reverse transcription reaction for cDNA synthesis was carried out in 20 µl reaction mixture using Revert Aid™ H Minus, Fermentas kit using Oligo dT primer according to the manufacturer’s protocols and reagents. cDNA samples were tested for the presence of *PaYMV* using potyvirus specific degenerate primers designed to amplify the coat protein gene. Forward primer (U335) 5′- GAATTCATGRTNTGGTGYATHGANAAYGG 3′ and reverse primer (D335) 5′-GAGCTCGCNGYYTTCATYTGNRHDWKNGC 3′ [28] respectively were used to amplify a fragment covering the cp gene. PCR was done using PCR kit by Invitrogen (Brazil). Thermal cycle was programmed as; first denaturation for 3 min. at 94°C was performed followed by 32 cycles of denaturation for 30 sec. at 94°C, 5 min of annealing at 60°C, extension for 30 sec. at 68°C and a final extension step at 68°C for 10 minutes. The PCR product was analyzed by electrophoresis in 1.2% agarose gel.

### 4.14. Cloning and Sequencing

The amplified fragments were cloned in pJET 1.2/blunt Cloning Vector using the PCR Cloning Kit, (Fermentas, USA) and transformed into XLI blue competent cells. Clones containing the PCR product of the expected size (based on primer locations) were identified by digestion with restriction enzyme –Bgl II and colony PCR (data not presented). This restriction site was present at both corners of blunt ends where insert was ligated and visualized by agarose gel electrophoresis and isolated by modified alkaline lysis method [29]. Sequencing was performed in automated sequencer (ABI Prism 310) with pJET1.2 forward sequencing primer 5′-CGACTCACTATAGGGAG AGCGGC-3′ using the Sanger’s dideoxy chain termination method [30].

### 4. 15. Pairwise Sequence Alignment for Similarity/Homology Studies

Closely related homologs were identified by pairwise sequence alignment studies. The partial coat protein gene sequences of Patchouli virus were compared with non-redundant database sequences of nucleotides and proteins by using Basic Local Alignment Search Tool (BLAST) program [31]. The database sequences were retrieved through GenBank database (NCBI, USA) (www.ncbi nlm.nih.gov/) [32].

### 4.16. Multiple Sequence Alignment for Phylogenetic Studies

After BLAST analysis, a data set of potential homologs was prepared by considering those database sequences which had >98% sequence identity to the query sequence. Phylogenetic analysis and nucleotide/protein conservation of the data set sequences were studied through multiple sequence alignment program *viz*., ClustalW v2.1 (http://www.ebi.ac.uk/clustalw) [33]. Genetic distances between the strains were calculated using the neighbor joining method. A phylogenetic analysis of Patchouli virus coat protein gene was performed to determine how the sequences of Patchouli virus and the other closely related virus strains might have been derived during evolution. The evolutionary relationships among the sequences were depicted by placing them as outer branches on a phylogenetic tree. The branching relationships in the inner part of the tree reflected the degree to which different sequences were related. Sequences that were very much alike were located as neighboring outside branches and joined to a common branch beneath them. The object of phylogenetic study was to find out all of the branching relationships in the tree along with branch lengths. For this, dendrogram and phylogram were constructed using distance method of phylogenetic tree construction. Distances between the studied sequences helped in understanding the evolutionary distances among the virus isolates.
